# Tailored exercise management (TEMPO) versus usual care for people aged 80 years or older with hip/knee osteoarthritis: study protocol for a feasibility randomised controlled trial

**DOI:** 10.1186/s40814-023-01275-5

**Published:** 2023-04-01

**Authors:** Philippa J. A. Nicolson, Melanie A. Holden, Ioana R. Marian, Esther Williamson, Susan J. Dutton, Angela Garrett, Sally Hopewell, Sarah E. Lamb

**Affiliations:** 1 Centre for Rehabilitation Research, Nuffield Department of Orthopaedics, Rheumatology and Musculoskeletal Sciences (NDORMS), , University of Oxford, Oxford, UK; 2 Primary Care Centre Versus Arthritis, School of Medicine, Keele University, Keele, UK; 3 Oxford Clinical Trials Research Unit, Centre for Statistics in Medicine, Nuffield Department of Orthopaedics, Rheumatology and Musculoskeletal Sciences (NDORMS), University of Oxford, Oxford, UK; 4 College of Medicine and Health, University of Exeter, Exeter, UK

**Keywords:** Over 80-year-olds, Osteoarthritis, Comorbidities, Exercise, Rehabilitation, Adherence

## Abstract

**Background:**

Exercise is recommended for all people with osteoarthritis. However, these recommendations are based on randomised clinical trials including people with an average age between 60 and 70 years, and these findings cannot reliably be generalised to people aged 80 years or older. Rapid loss of muscle occurs after 70 years of age, and older people are more likely to also have other health conditions that contribute to difficulties with daily activities and impact on their response to exercise. To improve care for people aged 80 or older with osteoarthritis, it is thought that a tailored exercise intervention targeting both osteoarthritis and any other health conditions they have, may be needed. The aim of this study will be to test if it is possible to conduct a randomised controlled trial (RCT) for people over 80 years of age with hip/knee osteoarthritis of a tailored exercise intervention.

**Methods:**

A multicentre, parallel, 2-group, feasibility RCT with embedded qualitative study, conducted in ≥ 3 UK NHS physiotherapy outpatient services. Participants (*n* ≥ 50) with clinical knee and/or hip osteoarthritis and ≥ 1 comorbidity will be recruited by screening referrals to participating NHS physiotherapy outpatient services, via screening of general practice records and via identification of eligible individuals from a cohort study run by our research group. Participants will be randomised (computer-generated: 1:1) to receive either: a 12-week education and tailored exercise intervention (TEMPO); or usual care and written information. The primary feasibility objectives are to estimate: (1) ability to screen and recruit eligible participants; (2) retention of participants, measured by the proportion of participants who provide outcome data at 14-week follow-up. Secondary quantitative objectives are to estimate: (1) participant engagement assessed by physiotherapy session attendance and home exercise adherence; (2) sample size calculation for a definitive RCT. One-to-one semi-structured interviews will explore the experiences of trial participants and physiotherapists delivering the TEMPO programme.

**Discussion:**

Progression criteria will be used to determine whether a definitive trial to evaluate the clinical and cost-effectiveness of the TEMPO programme is considered feasible with or without modifications to the intervention or trial design.

**Trial registration:**

ISRCTN75983430. Registered 3/12/2021. https://www.isrctn.com/ISRCTN75983430.

**Supplementary Information:**

The online version contains supplementary material available at 10.1186/s40814-023-01275-5.

## Background

Osteoarthritis (OA) of the hip and knee is the 11th leading cause of disability worldwide [1], and the most common joint disease in the United Kingdom (UK) [2]. The prevalence of hip and knee OA has a strong association with increasing age [3]. In the UK alone, approximately 3 million people were aged 80 or older in 2018, and this group is projected to increase to almost 6 million by 2043, making it the fastest growing population group [4]. Adults aged 80 years or older are the least physically active and have the highest healthcare expenditure [5, 6].

The presence of comorbidities is also increasingly common with age, with over 80% of people aged 85 years or older having two or more chronic conditions [7]. It has been suggested that management of patients with multiple conditions is now the most important task facing health services in developed countries [8]. The most common conditions among people with hip/knee OA include cardiovascular diseases, depression, type 2 diabetes, hypertension and other sites of musculoskeletal pain [9–11].

Current evidence-based guidelines recommend exercise for all patients with hip/knee OA, regardless of age, pain levels, disease severity, or functional ability [2, 12–14]. However, these recommendations are based on data from randomised trials commonly conducted in patients aged between 60 and 70 years [15, 16]. In Cochrane reviews of exercise for hip [16] and knee OA [15], no trials involved participants with an average age over 80. Between 70 and 80 years of age, many physiological changes occur, and people have a broad range of abilities at this age. Muscle strength declines 10 to 15% between 60 and 70 years of age, after which the loss accelerates to 25 to 40% between 70 and 80 years of age [17, 18]. In addition, endurance capacity declines approximately 10% between 70 and 80 years of age [19]. Given these changes, exercise recommendations from younger populations cannot reliably be generalised to adults aged 80 or older. Comorbidities may also interfere with the use of exercise among people with hip/knee OA. Current OA guidelines do not offer specific recommendations for comorbidity-associated exercise adaptions.

In clinical practice, over 42% of people aged over 75 years in the UK consult a General Practitioner (GP) regarding their OA [20], but they are significantly less likely to be referred for exercise than younger people [21]. This may reflect uncertainty around exercise prescription for this patient group, particularly in the presence of comorbidities [22], lack of existing suitable exercise interventions or settings, or reluctance from patients to engage in exercise [23].

The UK National Institute for Health and Care Excellence (NICE) have identified treatments for OA in very old people as a research priority, specifically the acceptability, nature, and setting for exercise strategies for this population [2]. NICE guidelines, and the 2019 Copenhagen Consensus statement on physical activity and ageing also highlight the need for tailored strategies that consider comorbidities in any approach to exercise for older people [24, 25].

The overall aim of the ‘Tailored Exercise Management for People aged 80 years or older with hip/knee Osteoarthritis’ (TEMPO) trial is to evaluate whether it is feasible to conduct a definitive multicentre RCT to test the clinical and cost-effectiveness of a tailored exercise intervention, compared to usual care, in improving functional status in adults aged 80 years and over with hip/knee OA and comorbidities.

Specifically, our primary objectives are to assess participant recruitment to, and retention in, the trial. Key secondary objectives are to determine participant engagement with the study and feasibility of the interventions from participant and physiotherapist (intervention provider) perspectives; to estimate the sample size calculation for a definitive trial, and to determine experiences and perceptions of the study design and intervention from both participant and physiotherapist perspectives.

## Methods/design

### Trial design

**Fig. 1:**
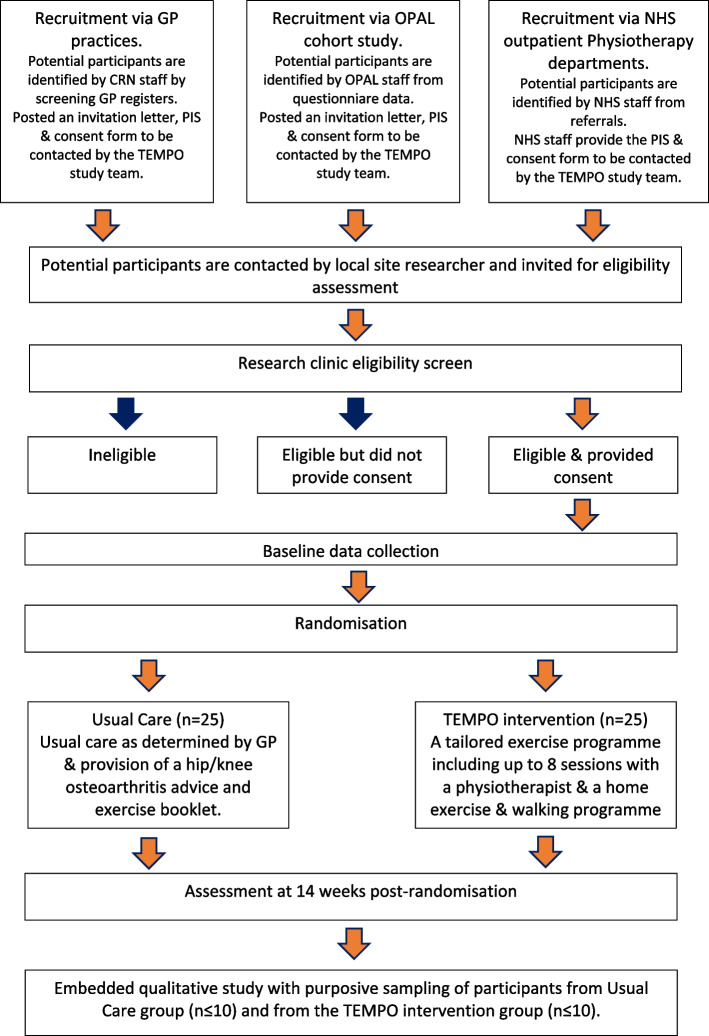
Study flow chart

A multicentre, parallel, 2-group, feasibility randomised (1:1) controlled trial with embedded qualitative study (outlined in Fig. 1). A schedule of enrolment, interventions, and assessments is presented in Table 1.

**Table 1:** Schedule of enrolment, interventions, and assessments for the TEMPO study

	Pre-randomisation	Baseline Assessment	Intervention period (0–14 weeks)	14-week Assessment	16–18 weeks after randomisation
Enrolment					
Potential participant identification and screening	X				
Provision of PIS	X				
Eligibility assessment	X				
Informed consent	X				
Assessment					
Demographics		X		X	
Nottingham Extended Activities of Daily Life Scale (NEADL)		X		X	
EuroQol EQ-5D-5L		X		X	
Average, worst and walking hip/knee pain (0–10 NRS)		X		X	
Nordic pain questionnaire		X		X	
Geriatric Depression Scale (GDS-15)		X		X	
Geriatric Anxiety Scale (GAS10)		X		X	
ProFANE self-report of falls and fall related injuries		X		X	
Walking confidence: single item from Modified Gait Self-Efficacy Scale		X		X	
Short Physical Performance Battery (SPPB)		X		X	
Current medications		X			
Attendance-treatment log (TEMPO intervention)			X		
Adherence-home exercise diary (TEMPO intervention)			X		
Adverse events recorded			X	X	
Additional treatments received				X	
Postal/phone collection of core outcomes (if required)					X
Qualitative interviews with sample of participants					X
Interventions					
Provision of hip/knee OA booklet (usual care)		X			
Referral to TEMPO physiotherapist		X			
TEMPO programme: 4–8 individual sessions with physiotherapist			X		

### Setting

Potentially eligible participants will be identified from referrals to participating NHS physiotherapy outpatient services, via screening of general practice records and via identification of eligible individuals from a cohort study run by our research group [26]. Interventions will be delivered within physiotherapy outpatient services at a minimum of three NHS sites across England.

### Eligibility criteria

The target population is adults aged 80 years or older with clinical knee and/or hip OA and at least one comorbidity. Full eligibility criteria are listed in Table 2.

**Table 2:** Eligibility criteria

Inclusion criteria
Registered with a primary care practice
≥ 80 years of age
Knee and/or hip osteoarthritis. GP register check (hip/knee osteoarthritis diagnosis recorded) or self-report of knee or hip joint pain lasting 3 months or longer AND knee or hip joint pain on most days of the past month
≥ 1 comorbidity. GP register check and/or self-report
Individual is willing and able to give informed consent for participation in the study
Exclusion criteria
Has a terminal condition with a life expectancy of less than 6 months or under palliative care
Any substantial health or social concern that, in the opinion of the inidividual’s GP, would place the individual at increased risk or inability to participate including known inability to provide informed consent
Significant cognitive impairment. GP check and/or assessed by research clinician. Eligibility screen includes the 6-item Cognitive Impairment Test (score of 8 or more are ineligible)
Unable to walk 3 m with or without an aid
Presents with signs of serious pathology requiring immediate referral for investigations
Unable to follow verbal or written instructions including inability to follow simple safety instructions

We have chosen to include participants with any comorbidities. Exercise adaptions are included in the intervention for comorbidities that we identified were both common among people with hip/knee OA and had a significant impact on function and/or mobility. We will assess which comorbidities participants report, and for the definitive trial will consider if additional comorbidity adaptions are required, or if participants with only specified comorbidities should be included.

### Participant identification

Three methods will be used to identify potential participants, informed by evidence that this population are often not referred to physiotherapy [21].

#### Screen of physiotherapy referrals

Physiotherapy referrals received by the NHS outpatient sites who will be providing the trial assessments and intervention delivery will be screened to identify adults aged 80 years and older with hip or knee pain potentially due to OA.

#### Identification of general practice consulters

We will work with the Clinical Research Network (CRN) to identify GP practices who have existing referral pathways to the NHS sites who will be providing the trial assessments and intervention delivery who are willing to take part in identifying and inviting potential participants. Electronic records of participating general practices will be screened to identify adults aged 80 years and over who have consulted a GP with knee and/or hip pain consistent with OA in the previous 36 months, and who have at least one other comorbidity, using Read/Systematized Nomenclature of Medicine (SNOMED) codes. The participating GP will screen the list and exclude any individuals who have any substantial health or social concern that, in their opinion, would place the individual at increased risk or inability to participate.

#### Screening of existing cohort study

The Oxford Pain, Activity and Lifestyle (OPAL) study is a prospective longitudinal cohort study of community-dwelling older adults aged 65 years or older recruited from 35 general practices across England. A detailed profile is published [26]. OPAL participants from GP practices who have existing referral pathways to the NHS sites who will be providing the trial assessments and intervention delivery who meet the inclusion criteria and have given permission to be contacted about participating in other studies will be identified and invited by the OPAL study team.

### Screening

Individuals who have been identified as potentially eligible to participate in the trial from one of the three methods described above will be mailed a covering letter and the Participant Information Sheet (PIS). If interested in participating, they will be asked to return a registration of interest form. A member of the trial team will then telephone the potential participant to check initial eligibility and answer any questions. If the person is potentially eligible, and, if they are willing to proceed, the researcher will invite them to a research clinic assessment at their closest participating NHS site.

#### Confirmation of eligibility assessment

Confirmation of eligibility will be undertaken at the research clinic assessment, by an experienced musculoskeletal physiotherapist. This assessment will include the 6-item Cognitive Impairment Test and red flag screening. The 6-item Cognitive Impairment Test is a brief screening tool for cognitive impairment, recommended for use in primary care [27]. Scores of 0–7 are considered normal and ≥ 8 significant. Participants who score ≥ 8 will not be eligible to participate in the trial, and their GP will be notified of the finding. Red flag screening will assess for signs of serious pathology requiring immediate referral for investigation.

### Consent

Prior to any study-related procedures or data being collected, written informed consent will be obtained. Permission from the participants will also be obtained to inform their GP of their inclusion in the study. The person who obtains the consent will be suitably qualified and experienced and have been delegated to do so by the site Principal Investigator.

### Randomisation, blinding, and allocation concealment

Following consent, participants will be randomised to the TEMPO intervention or Usual Care in a 1:1 ratio using the centralised web-based randomisation service provided by the Oxford Clinical Trials Research Unit (OCTRU). Randomisation will be stratified by centre. Randomisation will be completed in real time within the research clinic appointment after baseline data has been obtained. On randomisation of a participant, the central trial team and main site contact will be notified via an automated email.

By virtue of the design, it is not possible to blind participants or the physiotherapists delivering the intervention. The local researcher clinicians conducting the assessments will not be blinded as they will undertake randomisation, arrange referral to the TEMPO intervention and provide the usual care information leaflet. The trial statistician who will be performing analysis of the data collected will also not be blinded to treatment allocation.

### Interventions

#### Comparator: usual care

Treatment for participants in the usual care group will vary depending on their method of identification. Participants randomised to the usual care intervention who are identified via existing referrals to NHS physiotherapy outpatient departments will be treated as usual by a physiotherapist who has not been trained in the TEMPO intervention. Participants randomised to the usual care intervention who are identified by screening of GP records, or screening of participants in the OPAL cohort study will continue to be managed by their GP. Participants in the usual care intervention will be given an educational booklet produced by Versus Arthritis [28, 29] at the conclusion of their baseline research clinic assessment.

#### TEMPO intervention

The TEMPO intervention was developed over three phases, described in detail elsewhere [30]. Phase 1 included a systematic review of existing exercise interventions for community-dwelling adults aged 80 years and older, collating relevant condition-specific exercise guidelines and identifying which comorbidities to tailor the intervention to. Phase 2 involved qualitative interviews to identify barriers and facilitators to exercise, and exercise preferences among this population. Identified barriers and facilitators were mapped to the Theoretical Domains Framework (TDF). Phase 3 involved utilising the Theories and Techniques Tool to select the most effective behaviour change techniques (BCTs) to include in the intervention which linked to the relevant domains of the TDF.

**Fig. 2:**
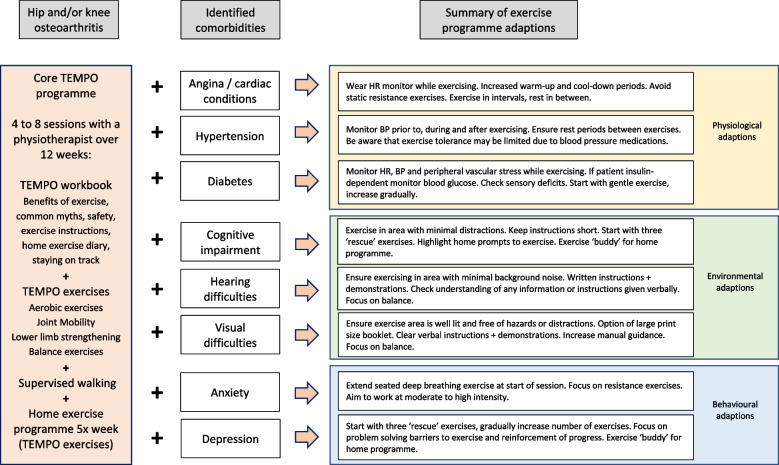
TEMPO intervention summary

The TEMPO intervention has four components: an evidence-based education workbook, a progressive exercise programme, a supervised walking programme, and a home exercise programme (Fig. 2).

Participants will have a minimum of four and maximum of eight one-to-one sessions with a physiotherapist in the outpatient physiotherapy department of the participating NHS trust over 12 weeks. Qualitative work undertaken in phase 2 of development found a strong preference from this population for individual sessions rather than group sessions. Interviewees felt that individual sessions allowed better tailoring of the intervention to their health conditions and ability and gave them confidence to exercise. The first session will be 60 min in duration and all other sessions will be 30 min. Session 1 will include a comprehensive assessment prior to commencing the TEMPO programme. Sessions one to four will be conducted face-to-face. Sessions 5 to 8 will be at the discretion of the physiotherapist and the participant, and may be delivered in-person, via video consultation or telephone call.

#### TEMPO workbook

Participants in the TEMPO intervention will be given a workbook which will include information covering the role of exercise for hip/knee OA, common myths about exercise for OA, the benefits of exercise for common comorbidities and safety when exercising. Participants will set a goal with the physiotherapist related to outcomes of the exercise programme within the intervention period and will complete a home exercise action plan. The workbook will include exercise demonstration photos and instructions, and prompt cards to remind participants to complete the exercises. An exercise diary will be included.

#### TEMPO exercise programme

The TEMPO exercise programme consists of aerobic, joint mobility, lower limb strengthening and balance exercises, targeted to functional activities such as getting out of a chair, walking, and climbing stairs. The physiotherapist will select which exercises are most appropriate for the participant based on their holistic baseline assessment and will tailor the intensity and level of each exercise based on the participant’s physical capability and the presence of comorbidities. We have classified comorbidity adaptions as physiological; environmental and behavioural. Participants may have adaptions for multiple comorbidities, and which adaptions are made is at the discretion of the physiotherapist. Exercises will be progressed over the course of the programme, by increasing the number of repetitions, by adding weight, or by increasing the speed with which the movement is completed.

Sufficient intensity of aerobic and strengthening exercises is essential in order to increase aerobic capacity and muscle strength [31, 32]. We will ask participants to initially work at a “moderate to somewhat hard” level of resistance for the aerobic and strengthening exercises so sufficient doses are achieved. This will be done by monitoring their responses using the Borg CR-10 Rating of Perceived Exertion (RPE) scale [33] which has been demonstrated as being a valid and reliable measure of exertion in older adults [34–36].

#### Supervised walking

Participants will undertake supervised walking with the physiotherapist if appropriate. Due to time constraints within a 30-min appointment the focus will be on challenging proprioception and balance rather than increasing walking distance.

#### Home exercise programme

Up to four of the exercises carried out during the physiotherapy sessions will constitute the home exercise programme, which participants will be asked to complete on 5 days each week. Participants will be supplied with the equipment required to complete the exercises. The number of sets and repetitions of home exercises will be set to ensure sufficient dose for physiological benefit, and we will ask participants to complete aerobic and strengthening home exercises at a “moderate to somewhat hard” level of resistance.

### Study training

Physiotherapists delivering the TEMPO intervention will be Band 6 or above, working in musculoskeletal outpatient departments in the NHS. Physiotherapists will attend a 3-h training session which will cover all aspects of the trial and processes involved and will receive a TEMPO manual. TEMPO intervention provision will be recorded by physiotherapists using case report forms (CRFs), which will be monitored and used to assess treatment fidelity.

### Concurrent healthcare for all participants

All participants will be advised that they can continue to access usual healthcare, including medications and consultations with other health professionals. Details of cointerventions will be recorded at the 14-week assessment.

### Primary feasibility outcomes

#### Participant recruitment

To estimate the recruitment achievable in a definitive trial we need to establish the recruitment and rate. The number of potential participants who are screened, eligible, consented, and randomised will be recorded on screening logs. Screening logs will also identify reasons that potential participants do not want to take part.

#### Participant retention

To estimate the rate of loss to follow-up in a definitive trial we will analyse the proportion of participants who provide outcome data at 14-week follow-up from those randomised.

### Secondary feasibility outcomes

#### Feasibility of interventions

Participant and physiotherapist engagement with the study and feasibility of the interventions will be assessed using a combination of quantitative and qualitative measures:

##### Fidelity

(1) The number of therapy sessions provided. Compliance with the protocol will be defined as delivery of ≥ four sessions, (2) Whether all sessions are delivered within 12 weeks of the first session, (3) The proportion of intervention components delivered within each session, (4) Whether any non-protocol treatments are delivered (e.g. manual therapy, taping), (5) Qualitative interviews with physiotherapists delivering the TEMPO intervention will explore the feasibility of delivering the intervention as per the protocol.

##### Participant exercise adherence

Proportion of participants that have received the TEMPO intervention reporting performance of home exercises ≥ three out of the five times per week prescribed (measured by home exercise diary completion).

##### Content of the usual care intervention

Details of treatments received will be recorded by the Research Clinician at 14-week Research Clinic Assessment.

### Acceptability of the interventions and study design

Qualitative interviews will explore participant and physiotherapist perspectives of the study design, including recruitment methods, study procedures, timeframes, and clinical outcomes. Findings from these interviews will guide any necessary modifications to study design and methods for the definitive trial.

### Secondary exploratory outcomes

We will collect a range of clinical outcomes at baseline and 14-week Research Clinic Assessments to determine their viability, and to inform the sample size calculation for the future definitive RCT (see Table 1 for summary of outcomes): self-reported function (Nottingham Extended Activities of Daily Life Scale (NEADL) [37]), health-related quality of life (EuroQol Group 5-Dimension Questionnaire (EQ-5D-5L) [38–40], average, worst and walking hip or knee joint pain (11-point Numeric Rating Scale), Geriatric Depression Scale [41], Geriatric Anxiety Scale [42, 43] and Prevention of Falls Network Europe (ProFANE) self-report of falls and fall related injuries. Research-clinician-observed functional status will be assessed (Short Physical Performance Battery (SPPB) [44, 45]).

### Adverse events

Adverse events (AEs) related to the trial intervention will be recorded and assessed using CRFs and contact with the trial team. Given the age range of the participant population and the nature of physical interventions, foreseeable AEs include acute infections (e.g. viral); medical instability (e.g. diabetic control—becomes hypoglycaemic, deterioration in control of heart failure); vestibular disorders and stroke; fall-related injuries; delayed onset of muscle soreness (≤ 72 h).

A serious adverse event (SAE) is an untoward medical occurrence that: results in death; is life-threatening; requires inpatient hospitalisation or prolongation of existing hospitalisation; results in persistent or significant disability/incapacity; consists of a congenital anomaly or birth defect. Where an SAE is potentially related to trial procedures, reporting procedures will be followed that are in accordance with Good Clinical Practice (GCP) guidance.

Safety reporting will begin from the time of randomisation and end when the participant has completed their 14-week follow-up Research Clinic Assessment.

### Sample size determination

As this is a feasibility study which is not aimed to assess treatment effects, we have not undertaken a formal power sample size calculation. A minimum of 50 participants will be recruited, based on Teare et al.’s [46] recommendation that between 50 and 70 are required when continuous scale data outcomes are to be collected. Achieving this sample size will require an average estimate of 2 participants per month per site, over the 9-month recruitment period. This sample size will also provide sufficient data to answer our feasibility objectives. Outcome data for the NEADL will be used to estimate standard deviations and CIs of the treatment estimates which will be used to inform a sample size calculation for a definitive trial.

### Data collection

#### Baseline research clinic assessment

Participants will complete a self-reported paper questionnaire booklet at the baseline Research Clinic Assessment (see Table 1 for summary of questionnaire content). The research clinician will assess height and weight, current medications and the Short Physical Performance Battery (SPPB).

#### Treatment logs

For all TEMPO intervention sessions, the date, clinician details, session number, mode of delivery, duration, session content, materials, and resources issued will be recorded on paper treatment logs.

#### 14-week research clinic assessment

All participants will attend an in-person 14-week assessment if possible. At this assessment, participants will complete a self-reported questionnaire booklet (see Table 1 for summary of questionnaire content). The Research Clinician will assess the Short Physical Performance Battery (SPPB) and record details of any cointerventions.

If a participant is unable to attend this assessment, then they will be posted the questionnaire, but we will be unable to carry out a physical assessment. If the questionnaire is not received by the central TEMPO study team within one calendar month of posting, or if the questionnaire is returned with missing data for the NEADL, a TEMPO researcher will telephone the patient participant, and if they are agreeable will complete the NEADL over the telephone.

### Early discontinuation/withdrawal of participants

During the trial, all participants have the right to withdraw at any time. In addition, site researchers may discontinue a participant from the study intervention if they consider it necessary for any reason. Participants who withdraw from the study or whose participation in the study is discontinued will have data collected up to the point of that withdrawal included in the analyses, unless the participant specifically asks for all data collected to be destroyed. Withdrawn participants will not be replaced.

### Progression criteria

Progression criteria to assess feasibility of a future definitive trial will be assessed using a traffic light system for quantitative feasibility outcomes [47] (Table 3). These quantitative progression criteria will be considered in combination with qualitative findings to guide decision making and trial design. ‘Green’ indicates feasible with current procedures, ‘Amber’ indicates modification to one or more components of the protocol is required to proceed, and ‘Red’ indicates a definitive trial would not be considered feasible.

**Table 3:** Quantitative progression criteria for the TEMPO study

Criteria	Green (Go)	Amber (Amend)	Red (Stop)
Recruitment	At least 50 eligible participants are identified and agree to take part over 9 months	Between 35 and 49 eligible participants are identified and agree to participation over 9 months	< 35 eligible participants are identified and agree to participation over 9 months
Consent	At least 40% of eligible potential participants consent to be randomised	20–39% of eligible potential participants consent to be randomised	< 20% of eligible potential participants consent to be randomised
Proportion of randomised participants providing 14-week outcome data^a^	At least 85% of participants provide outcome data at 14 weeks	60 to 84% of participants provide outcome data at 14 weeks	< 60% of participants provide outcome data at 14 weeks
Intervention fidelity	At least 75% of participants receive the allocated intervention sessions as per protocol (minimum of 4 sessions delivered over 12 weeks)	50 to 74% of participants receive the allocated intervention sessions as per protocol (minimum of 4 sessions delivered over 12 weeks)	< 50% of participants receive the allocated intervention sessions as per protocol (minimum of 4 sessions delivered over 12 weeks)

^a^Follow-up would be at least 6 months in the definitive RCT so this is a proxy

### Data management

Data will be collected using paper CRFs. All data will be sent to the central TEMPO team at the University of Oxford for entry into Research Electronic Data Capture (REDCap). Contact details will be stored separately from the outcome data and will be deleted when no longer required as part of the study (within 12 months after the last data collection). All data will be handled and stored in line with Oxford Clinical Trials Research Unit Data Security procedures and Standard Operating Procedures which are in accordance with the Data Protection Act 2018, other relevant regulations and GCP guidelines.

### Statistical methods

The primary analysis will evaluate the feasibility of conducting a future definitive multicentre RCT. Descriptive statistics of the following feasibility outcomes will be reported: recruitment screening logs, consent forms, and logs of data collection forms; TEMPO intervention session attendance, home exercise adherence, treatment/s received by usual care group, attrition rate, safety reporting forms; follow-up participant questionnaire (and clinical assessment) completion rates of those randomised.

Outcome measures will be reported descriptively and differences between treatments for the Intention To Treat (ITT) population will be reported together with 95% confidence intervals (CI). Withdrawals from treatment or the trial, related AEs and SAEs will be reported. Mean and standard deviation or median and interquartile range will be used for continuous variables and counts, and numbers and percentages will be used for any binary or categorical variables. Missing data will be minimised by careful data management. No comparative statistical testing will be undertaken as the study is not powered for this purpose.

Additional analyses will evaluate the proposed primary and secondary outcomes planned for the full trial. The planned primary outcome for the full study, NEADL, will be measured and reported at baseline and 14-week follow-up for each intervention group. The difference in the means between the intervention group compared to usual care together with the corresponding 95% CI will be reported. Analysis of the primary outcome will be performed using multivariate linear regression with adjustment for the baseline NEADL score, number of comorbidities reported and stratification factor: centre. Analysis methods for the primary outcome will be conducted for secondary outcomes, with logistic regression being used for binary outcomes and linear regression for continuous ones. No statistical tests will be performed as the study would not be powered to detect any differences.

### Embedded qualitative study

An embedded qualitative study will assess the feasibility and acceptability of the interventions from the perspectives of those delivering (physiotherapists) and receiving (participants) the interventions, and their experiences of taking part in the study. Qualitative outcomes will guide modifications to the content and delivery of a future definitive trial.

We will undertake semi structured interviews (via telephone or face to face) with up to 20 participants (up to 10 from each treatment arm) within 4 weeks of completing their 14-week follow-up assessment. Participants who have agreed to be contacted for the interview will be purposively sampled to ensure demographic representation of the full study sample. Targeted demographics include age, ethnicity, hip or knee OA, number of comorbidities and baseline functional level. The interview topic guide will seek to explore participant opinion and experience of study recruitment, intervention content, timing, and accessibility and barriers and facilitators to adherence.

All physiotherapists who deliver the TEMPO intervention will be invited to be interviewed. The interview topic guide will cover the experience of trial training, views on the TEMPO intervention content, their role within the trial, and delivery of the intervention.

All interviews will be audio recorded, independently transcribed verbatim, checked, and anonymised. Transcriptions will be managed using NVIVO software [48]. Data will be analysed using thematic analysis, following the six steps proposed by Braun and Clarke [49].

### Data monitoring

This study will be coordinated by the UK Clinical Research Collaborative registered Oxford Clinical Trials Research Unit (OCTRU) at the University of Oxford. A rigorous programme of quality control will be implemented to ensure compliance to the current approved protocol, GCP, relevant regulations and OCTRU Standard Operating Procedures (SOPs). The Chief Investigator and the Senior Trial Manager will develop data management and monitoring plans. The day-to-day management of the trial will be the responsibility of the Chief Investigator, overseen by the Trial Management Group (TMG), who will meet monthly to assess progress. Quality assurance checks will be undertaken by the trial management team to ensure integrity of randomisation, study entry procedures, and data collection. A minimum of one inspection of the Trial Master File will be carried out by the OCTRU Quality Assurance team in the lifetime of the study. Intervention delivery will be monitored periodically to ensure fidelity. Site visits and/or audio/video recording of interventions will be conducted. Additionally, the study may be monitored, or audited by sponsor or host sites in accordance with the current approved protocol, GCP, relevant regulations, and standard operating procedures.

### Patient and public involvement

A patient and public involvement (PPI) group consisting of eight members was established when intervention and trial development work began. The selection of clinical outcome measures was guided by the preferences of the PPI group. Feedback from the group determined the mode of delivery of the intervention, the number of physiotherapy sessions, and content of the TEMPO workbook. A member of the PPI group is also a member of the TMG.

### Ethics and dissemination

This study was approved by the London-Brent Research Ethics Committee, ref: 21/LO/0777. This protocol has been reported following the Standard Protocol Items: Recommendations for Interventional Trials (SPIRIT) statement [50] (Completed SPIRIT checklist: Additional file 1).

Results will be published in a peer-reviewed journal with authorship eligibility according to the International Committee of Medical Journal Editors criteria. The final report will detail amendments to the study protocol. A plain language summary of the results will be posted to the trial participants. The findings will be shared with patients and the public more widely through local and national charity (e.g. versus arthritis) newsletters and other media channels. Social media will be utilised to share news on study progress.

### Trial status

The first participant was randomised to the trial on 30 May 2022. Recruitment is ongoing.

## Discussion

This randomised feasibility trial assesses a tailored exercise intervention, targeted to improve functional status in adults aged 80 years and over with hip/knee OA and comorbidities. The exercise programme is delivered with behaviour change techniques to facilitate engagement and regular home exercise by participants.

This feasibility trial has several strengths. The TEMPO programme was systematically developed according to Medical Research Council guidance, combining current best evidence, existing exercise guidelines, qualitative findings from interviews with participants and behaviour change theory. Development of the programme included significant input from patient and public representatives and clinicians who manage adults aged 80 years and over with hip/knee OA. Therapists who deliver the programme will undertake standardised training on the content and delivery of the programme, and trial methods and reporting. To our knowledge, this is the first trial to evaluate an exercise programme specifically designed for this population group. Our inclusion of both quantitative and qualitative data collection methods, and the embedded qualitative study including both participants and therapists will provide rich information about the feasibility of undertaking a large-scale RCT of the TEMPO programme.

The trial also has limitations. We are only able to undertake the trial at a limited number of sites, which will limit our ability to assess feasibility across the wider NHS. However, we have purposively recruited sites that represent a range of geographical and clinical settings, and who typically see patients from a range of socio-demographic backgrounds.

If this feasibility trial is successful, it will guide the development of a definitive RCT to test whether the TEMPO programme is clinically and cost-effective in the NHS.

## Supplementary Information

**Additional file 1.** SPIRIT checklist.

## Data Availability

Not applicable.

## Abbreviations

AE: Adverse event
CI: Confidence interval
CRF: Case Report Form
EQ-5D-5L: EuroQol Group 5-Dimension Questionnaire
GCP: Good Clinical Practice
GP: General practitioner
ITT: Intention to treat
NEADL: Nottingham Extended Activities of Daily Life Scale
NHS: National Health Service
NICE: National Institute for Health and Care Excellence
OA: Osteoarthritis
OCTRU: Oxford Clinical Trials Research Unit
OPAL: Oxford Pain, Activity and Lifestyle cohort study
PIS: Participant Information Sheet
PPI: Patient and Public Involvement
ProFANE: Prevention of Falls Network Europe
RCT: Randomised controlled trial
REDCap: Research Electronic Data Capture
SAE: Serious adverse event
SNOMED: Systematized Nomenclature of Medicine
SOP: Standard Operating Procedure
SPIRIT: Standard Protocol Items: Recommendations for Interventional Trials
SPPB: Short Physical Performance Battery
TEMPO: Tailored Exercise Management for People aged 80 years and older with hip/knee Osteoarthritis
TMG: Trial Management Group
UK: United Kingdom
